# Use of two vertical injectors in place of a horizontal injector to improve the efficiency and stability of THAI in situ combustion process for producing heavy oils

**DOI:** 10.1007/s13202-021-01345-5

**Published:** 2021-10-30

**Authors:** Muhammad Rabiu Ado

**Affiliations:** Department of Chemical Engineering, College of Engineering, King Faisal University, P.O. Box 380, Al-Ahsa, 31982 Kingdom of Saudi Arabia

**Keywords:** In situ combustion (ISC), Enhanced oil recovery (EOR), Bitumen/heavy oil/tar sand, Reservoir simulation, Toe-to-heel air injection (THAI), Thermal EOR

## Abstract

The current commercial technologies used to produce heavy oils and bitumen are carbon-, energy-, and wastewater-intensive. These make them to be out of line with the global efforts of decarbonisation. Alternative processes such as the toe-to-heel air injection (THAI) that works as an in situ combustion process that uses horizontal producer well to recover partially upgraded oil from heavy oils and bitumen reservoirs are needed. However, THAI is yet to be technically and economically well proven despite pilot and semi-commercial operations. Some studies concluded using field data that THAI is a low-oil-production-rate process. However, no study has thoroughly investigated the simultaneous effects of start-up methods and wells configuration on both the short and long terms stability, sustainability, and profitability of the process. Using THAI validated model, three models having a horizontal producer well arranged in staggered line drive with the injector wells are simulated using CMG STARS. Model A has two vertical injectors via which steam was used for pre-ignition heating, and models B and C each has a horizontal injector via which electrical heater and steam were respectively used for pre-ignition heating. It is found that during start-up, ultimately, steam injection instead of electrical heating should be used for the pre-ignition heating. Clearly, it is shown that model A has higher oil production rates after the increase in air flux and also has a higher cumulative oil recovery of 2350 cm^3^ which is greater than those of models B and C by 9.6% and 4.3% respectively. Thus, it can be concluded that for long-term projects, model A settings and wells configuration should be used. Although it is now discovered that the peak temperature cannot in all settings tell how healthy a combustion front is, it has revealed that model A does indeed have far more stable, safer, and efficient combustion front burning quality and propagation due to the maintenance of very high peak temperatures of mostly greater than 600 °C and very low concentrations of produced oxygen of lower than 0.4 mol% compared to up to 2.75 mol% in model C and 1 mol% in model B. Conclusively, since drilling of, and achieving uniform air distribution in horizontal injector (HI) well in actual field reservoir are costly and impracticable at the moment, and that electrical heating will require unphysically long time before mobilised fluids reach the HP well as heat transfer is mainly by conduction, these findings have shown decisively that the easy-and-cheaper-to-drill two vertical injector wells configured in a staggered line drive pattern with the horizontal producer should be used, and steam is thus to be used for pre-ignition heating.

## Introduction

There is no doubt that the corona virus pandemic has hugely affected the global energy and thus economic system. The effect might be felt for many years to come especially that it is yet to be fully under controlled. However, governments and businesses are adapting as the race to vaccinate all humans is on. As a result, economic activities together with use of oils will increase. Based on the analysis of the most recent International Energy Agency’s (IEA’s) projections about increase in oil demand, it is found that around 770 billion barrels of oil are needed from now to year 2040 (International Energy Agency 2020). However, the British Petroleum (BP) 2020 statistical review of world energy has shown that the total proved reserves of recoverable conventional and unconventional oils are 1734 billion barrels globally as at the end of 2019 (British Petroleum (BP) 2020). Out of that, a total of 70% of the 1734 billion barrels of oil are composed of unconventional oils (i.e. heavy oils and bitumen) (Elahi et al. 2019; Guo et al. 2016; Liu et al. 2019). This implies that conventional light oils make up the remaining 30% of the recoverable resources (i.e. 520.2 billion barrels). Putting all of the above together, if only all of the light oils are to be used, they are not enough to cater for the demand from now to the next two decades. Therefore, it cannot but be concluded that at least 249.8 billion barrels of heavy oils and bitumen are needed to satisfy the demand from now to the next 20 years. Furthermore, without new discoveries of recoverable light oils reservoirs, beyond 2040, all our petroleum consumption must come from the heavy oils and bitumen. This is so because still there is no alternative capable of meeting the increasing demand from petrochemical and transportation sectors. Also, plastics and other medical equipment must continuously be made especially in this age where virus is rapidly being transmitted throughout the world. Heavy oils and bitumen are very sticky and most of them do not flow under natural reservoir conditions due to their very high viscosity of up to 1.2 × 10^2^ cP and API gravity of even lower than 8° API depending on the reservoir (Attanasi and Meyer 2010; Hein 2017; Li et al. 2020; Meyer et al. 2007; Zhang et al. 2019). Thus, the urgent need for technologies for energy-efficient and environmentally friendly upgrading and production of heavy oils and bitumen cannot be overstated. However, it must be stressed that the current commercial steam-based extraction and production methods are carbon and energy intensive. More so, these processes do not permanently alter the physicochemical properties of the oils such that even if the produced oil is returned to the typical reservoir temperature, it should not loss its mobility due to increase in viscosity and density. Multiple studies have shown that the most popular steam-based process, the steam-assisted gravity drainage (SAGD), cannot be applied widely as production from certain reservoirs is uneconomical or unsafe or both (Gates 2010; Gates and Larter 2014; Ma and Leung 2020; Mokrys and Butler 1993; Paitakhti Oskouei et al. 2011; Shah et al. 2010; Shi et al. 2017; Turta and Singhal 2004; Zhao et al. 2013, 2014). Additionally, steam-based processes are water intensive as they handle very large quantity of water and wastewater. And in fact, some of the SAGD operations have been shown to not be net energy producers (Gates and Larter 2014). In-situ-combustion-type (ISC-type) processes hold considerable promise as they do not suffer from any disadvantages of the steam-based processes. However, it is worth pointing out at this stage that the operation of the ISC-type processes is highly complex because they are composed of simultaneous multiphase and multicomponent reactive transport systems in porous media.

Toe-to-heel air injection (THAI) is an in-situ-combustion-type process that uses horizontal producer (HP) well in combination with a horizontal injector (HI) to form HIHP configuration in a staggered line drive pattern or either a single vertical injector (VI) well to form VIHP arrangement in a direct line drive pattern or double vertical injectors (2VI) to form 2VIHP wells configuration in a staggered line drive pattern (Greaves et al. 1993; Greaves and Al-Shamali 1996; Xia et al. 2002). HIHP and 2VIHP configurations were respectively only used in laboratory experiments, whilst VIHP was used in both the laboratory experiments and field operations. Since the issue of well configurations is discussed briefly here, I shall discuss it in more details later since partly, it is one of the aims of this work. At experimental level, during start-up phase, different fluids (such as hot nitrogen or steam) or electrical heaters are used to prepare the inlet zone of the injector(s) so that enough heaviest fraction of the oil is left to be burnt as coke. Also, the pre-ignition heating is performed to establish fluids communication between the wells (Ado 2020a; Greaves et al. 1999; Rabiu Ado 2017; Xia and Greaves 2002, 2006). Study by Liang et al. (2012) has stressed the importance of optimum start-up so that the combustion front should always be maintained as forward leaning rather than back-slanting. Immediately the pre-ignition heating cycle (PIHC) is over, air injection commenced which is then followed by electrical ignition. Continual injection of air results in propagation and expansion of the combustion zone in all the three directions, leading it to become conical in shape with narrow end pointing downward towards toe of the HP well. In terms of the physicochemical processes, at least five distinct-property zones develop right from the combustion front up to the heel of the HP well. Ahead of the combustion front lies the coke zone where high-temperature oxidation (HTO) reaction which fuels the process takes place. There forward lies the thermal cracking zone in which coke is deposited and lighter components are produced which then mixed with mobile oil in it is zone (i.e. MOZ). Between thermal cracking zone and MOZ, a steam zone exists which is formed due to both vaporisation of reservoir native water and from the combustion gases. Ahead of MOZ lies the cold oil zone (COZ) where not enough heat has reached to alter flow-ability or physical properties of the oil in that zone (Rabiu Ado et al. 2017, 2018; Xia et al. 2005). Although the THAI process is largely far away from being understood as a result of the absence of general and reservoir-specific design procedures and operation manuals, laboratory experimental studies have consistently shown that the process’s recovery factors are up to 85.5% of oil originally in place (OOIP), that permanent physical and chemical alterations were achieved as shown in terms of increase in API gravity by up to an average of 10° points in addition to that of the original bitumen, and that viscosity reduction down to 50 mPas was achieved (Greaves et al. 1999; Xia and Greaves 2002, 2006; Xia et al. 2002). Other advantages of the THAI process as found experimentally and through numerical simulation include generation of large amount of hydrogen gas which is partly used for hydro-conversion and partly produced with the combustion gases, large quantity of sulphur and heavy metals are locked in the reservoir showing that the process is relatively environmentally friendlier, and since the process uses short-distance displacement mechanism to produce mobilised upgraded oil, realisation of return on investment will be very fast compared to in the other processes (Greaves et al. 2008; Turta and Singhal 2004; Xia et al. 2005). Another import merit of the THAI process is the ease with which an annular layer of hydro-treating catalyst can be added around the HP well so that apart from thermal cracking upgrading, additional upgrading is achieved catalytically. Ado et al. (2021) are the first to conduct multiple research on numerically simulating in situ combustion process coupled with in situ catalytic upgrading process to form a single process referred to as THAI-CAPRI.

At field scale, two THAI projects were operated by Petrobank (later called Touchstone). In the first pilot study, upgraded oil was produced albeit at smaller production rates. However, there was excessive sand production that considerably affected the economic performance of the process. Also, there was no direct relationship between injected air and produced oil (Petrobank 2007, 2008, 2009). The company considered the learning from the project a technical success despite their total failure to economically prove the process. Lack of comprehensive design and operation procedures are also part of the reasons why the technical validity of the process is only partial. Moving forward, the company started a semi-commercial operation in Kerrobert, Canada, where oil production rates were initially profitable before, may be, air channelling into the bottom water zone, or into the overlaying gas reservoirs or both, destabilised the combustion zone, leading to fingering and loss of control. This caused oil production rates to decrease to the extent that the company had to dispose of the project due to uncontrollable operating losses. Part of the reasons why healthily-structured combustion front was not established could be due to insufficient PIHC and/or improper design since their designs were based on reservoir simulations using kinetics that were not up-scaled and thus could well result in erroneous designs as has been found out by Ado ([Bibr CR2][Bibr CR3], d). These two Canadian projects have therefore validated the THAI process only partially technically. In terms of the process taking place as a major heavy oils and bitumen upgrading and producing technology, the economic validity of the process must be fully established. There are around three studies published in 2020 about the Kerrobert THAI project. All of them concluded to the following effect: there is an air injection rate limit beyond which no gain in incremental oil production rates can be obtained, and therefore, air injection rate must not be more than 20,000 Sm^3^/day (Wei et al. 2020a, b). However, this conclusion is very weak because numerical simulation studies, which are under review, have shown that air could be channelling into the bottom water zone thereby causing excessive water production (i.e. high water cuts) as observed by Turta et al. (2020). Also, there were shale inter-layers in the reservoir that might have caused some of the oxidant to be bypassing the combustion front and be moving into the upper permeable rock especially if it did not provide total effective sealing (Ado et al. 2019). Also, the large air injection rates could be part of the reasons as one of the pilot studies in China has shown that optimum air flux must lie between 0.15 and 0.3 Sm^3^/m^2^ h (Xi 2016) and still the combustion will be in an HTO mode. Ado (2020e) conducted numerical simulations and found that air injection flux must be kept to optimally minimum value of 0.35 Sm^3^/m^2^∙h, which is similar to the conclusion drawn by Wei et al. (2020b), to avoid channelling, fingering, and bypassing of combustion front. Also, Ado has shown that using pure oxygen as the injected oxidant results in large increase in cumulative oil recovery of at least 3.85% OOIP (Ado 2021). Apart from the Canadian THAI projects, Turta (2018) claimed that there are THAI pilot studies in China and in India. The information available about one of the projects in China indicated that oil production rates of up to 400 barrels per day are being achieved and that the process operates in HTO mode with very high volumetric sweep efficiency and recovery factor (Xi 2016). In all of these projects, Turta (2018) found that all the vertical injector/horizontal producer (VIHP) well pairs were or are configured in direct line drive (DLD) pattern. This implies that the VI well is on the same mid-plane as the HP well even though the former is offset axially and is located vertically above the latter. The DLD configuration is thought to not provide high oil production rates due to the inherently smaller volume of the combustion front. Also, DLD arrangement leads to premature oxygen production and eventually breakthrough. Since there are no conclusive studies about the best well configuration and how it will affect the stability of the combustion front and thus the oil production rates, the studies that attempted to provide the studies, albeit non-exhaustive, merit reviewing here.

The first experimental study of the effect of wells configuration on the performance of the THAI process was conducted by Xia et al. (2002). They concluded that HIHP is the best configuration for realising efficiently rapid start-up and achieving stable combustion front advancement despite the fact that they indicated that it is not a practical design in actual field operation and that they did not report about the stability of the process over the lifetime of the experiment. They also found that VIHP DLD pattern has the lowest recovery as a result of oxygen breakthrough resulting in early extinguishment of the combustion front. However, that study did not explain if in the long term, the stability achieved in the HIHP arrangement is sustained up to the end of operating time. Also, this study did not investigate the effect of start-up methods on the various wells configurations. That is to say, with VIHP arrangement, is injecting steam, hot nitrogen, or electrical heating during the PIHC going to provide and results in sustaining stable combustion front propagation throughout the duration of the experiment. Also, their study did not provide information about the performance of 2VIHP configuration both during start-up and over full operation period. Fatemi et al. (2009) performed numerical simulation studies to investigate the optimum well pairs configurations for the THAI process. They found that 2VIHP and VIHP are the best respective arrangement in terms of many performance indicators, such as oil rates, ultimate oil recoveries, produced combustion gases, and volumetric sweep. However, the authors conducted the study using electrical heating to condition the inlet zone of the 3D combustion cell and probably to establish fluid communication between the two wells. That means, they did not consider the fact that if another pre-ignition heating method is used, for example using steam, how are the different wells configurations going to affect the performance of the THAI process throughout the operation time (i.e. from start-up to shutdown). Furthermore, none of the two studies used steam for the PIHC despite the fact that it is the most abundant fluid that is used in heavy oils and bitumen mobilisation and production. One of the latest studies by Zhao et al. (2018) investigated about the influence of injector–producer spacing on the performance of the THAI process. They concluded that the spacing should be optimised for actual field operation design which is as expected. However, the study also used electrical heater to pre-heat the inlet zone of the VI well and subsequently achieve ignition. Steam, which is used in the field for PIHC, should have been used because the flow pattern and the shape of the combustion zone are partly determined by the method used for the PIHC. A recently published study by Ado (2021) has shown that comparatively, 2VIHP is far more efficient, stable, and profitable than VIHP configuration. However, that study did not check what will happen if a different PIHC method is used instead of steaming. Also, that study work did not operate the THAI process up to the cut-off point beyond which continual operation is either unsafe, uneconomical, or both.

From the above literature survey, none of the studies is conclusive, and thus, it cannot but be concluded that the THAI process is far from being fully understood in terms of start-up phase, operation phase, and shutdown stage. Here, stress is placed on the start-up phase because in most cases, if not all, it is the ultimate determiner of the success or otherwise of the THAI process. It then naturally follows that full understanding of optimal process design and operating conditions is necessary if the massive economical promise of the THAI process is to be fully realised. Thus, to move forward, design and operating manuals are essentially required as outlined in an unpublished review by Ado (2021). Additionally, from the above literature review and in order to develop a standard start-up procedure, it cannot but be concluded that the method of starting-up the operation of the THAI process must consider optimal operation by ensuring stable combustion initiation and its front propagation, safety, and profitable oil production rates are maintained initially and throughout the lifetime of the project. Thence, among the many process design factors that must be accurately and optimally predicted and set prior to full field deployment are the wells configurations and the method of pre-ignition heating. As a consequence, the aim of this work is to investigate the performance of the THAI process based on wells configurations and start-up methods, and it is stability over the lifetime of the operation by developing three numerical models based on a validated numerical model that was developed and simulated using computer modelling group (CMG) thermal reservoir simulator, STARS, which is selected because it is one of the major thermal reservoir simulators being used industrially and academically to investigate and predict performance of various thermal and even non-thermal enhanced oil recovery processes. These three models which are referred to as A, B, and C respectively and which are validated and reported in Ado (2020a), are:

A. Two vertical injectors/horizontal producer (2VIHP) wells pair model that saturated steam at 226 °C and 2600 kPa was used during its pre-ignition heating cycle (PIHC).
B. Horizontal injector/horizontal producer (HIHP) wells pair model that electrical heating at rate of 2115 J min^−1^ was used during the PIHC, and
C. Horizontal injector/horizontal producer (HIHP) wells pair model that saturated steam at 226 °C and 2600 kPa was used during the PIHC.

For model B, in accordance with the experiment (Xia and Greaves 2002), electrical heater/ignitor was used with heating rate in each of the 17 gridblocks of 35.25 W to heat the inlet zone of the sandpack, establish communication with the HP well, and initiate combustion. The pre-ignition electrical heating was conducted for 28 min and ignition was achieved over 2 min. For models A and C, steam containing the equivalent amount of heating rate in model B was injected at a rate of 21.17 cm^3^ min^−1^ cold water equivalent (CWE) for a period of 28 min in each of the two models. Then, in each model, electrical heater/ignitor was used for 2 min to further increase the temperature of the inlet zone of the sandpack and subsequently to initiate the combustion. More information about the method will be discussed under the methodology section.

This study has shown conclusively that the use of 2VI wells together with steaming during the PIHC is by far more efficient, safer, and more economical due to higher cumulative oil recovery. More so, since drilling of, and achieving uniform air distribution in horizontal injector (HI) well in actual field reservoir are costly and impracticable at the moment, and that electrical heating will require unphysically long time before mobilised fluids reach the HP well as heat transfer is mainly by conduction, these findings have shown decisively that the easy-and-cheaper-to-drill vertical injector wells arranged with HP well in a staggered line drive (SLD) configuration should be used in actual field reservoir.

## Numerical simulation models development

**Fig. 1:**
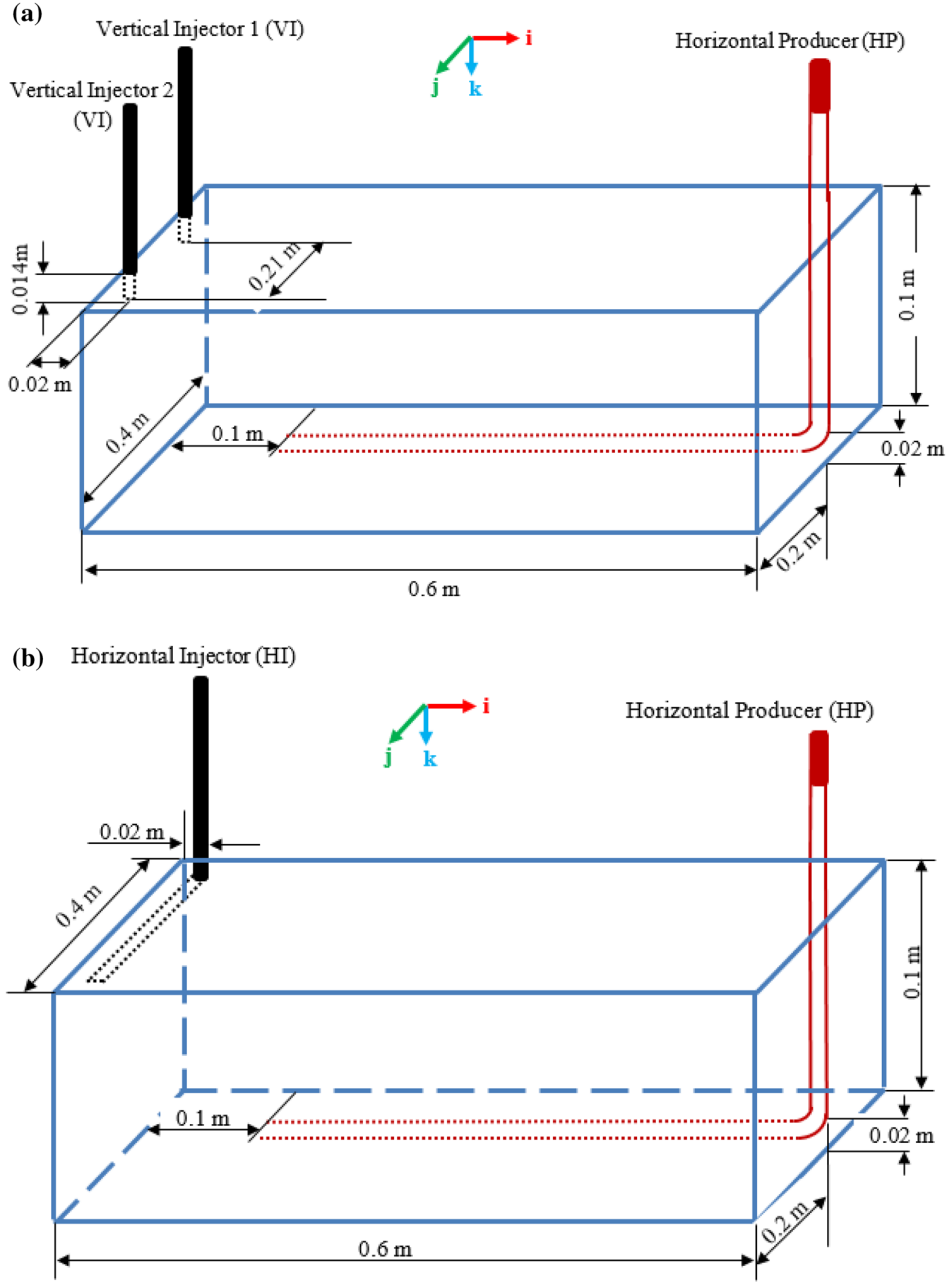
Three-dimensional laboratory-scale combustion cell showing the dimensions, wells configurations, and the cardinal directions of **i, j, & k**. **a** Shows 2VIHP arrangement in staggered line drive pattern, and **b** shows HIHP arrangement in staggered line drive pattern

In this work, ideal homogenous sandstone bottom-water-free Athabasca-bitumen-type reservoir is simulated at experimental scale. Therefore, the model reservoir parameters and properties are those of the Canadian Athabasca bitumen with exception of those parameters, such as absolute permeability, fluids saturations, temperature, and pressure, that depend on the sand packing and the laboratory. The numerical model dimensions are similar to that reported experimentally (Xia and Greaves 2002) and to the three different numerical models validated against the former (Ado 2020a). All the three numerical models were constructed using the computer group modelling (CMG) reservoir simulator, i.e. the CMG STARS. The models dimensions, wells configurations, and the number of gridblocks in which the reservoir is discretised into 30 in **i**^**th**^ direction by 19 in **j**^**th**^ direction by 7 in **k**^**th**^ direction are shown in Fig. 1. It is very important at this stage to specify that model A is the one shown in Fig. 1a, whilst models B & C are the ones shown in Fig. 1b. The difference between B & C is the use of electrical heating in the former and use of steam in the latter during the PIHC. This simulator allows discretised wellbore (DW) option to enable the dynamic nature of the multicomponent, multiphase fluid flow and heat transport to be numerically simulated after discretisation. The DW, which represents the HP well, and discretised reservoir reactive transport equations are then solved simultaneously by the use of parallel processing solver called PARASOL. Each model was run on a computer that has 16 cores and 32 threads. However, the STARS allows only 4 cores for the parallel computation, and therefore, only 25% of the threads (i.e. 8 threads) were used. Onwards, the simulation cannot be run without fully defining the boundary and initial conditions. Also, operating conditions and reservoir properties, such as porosity and permeability, etc., must be specified.

### Reservoir petro-physical parameters

**Fig. 2:**
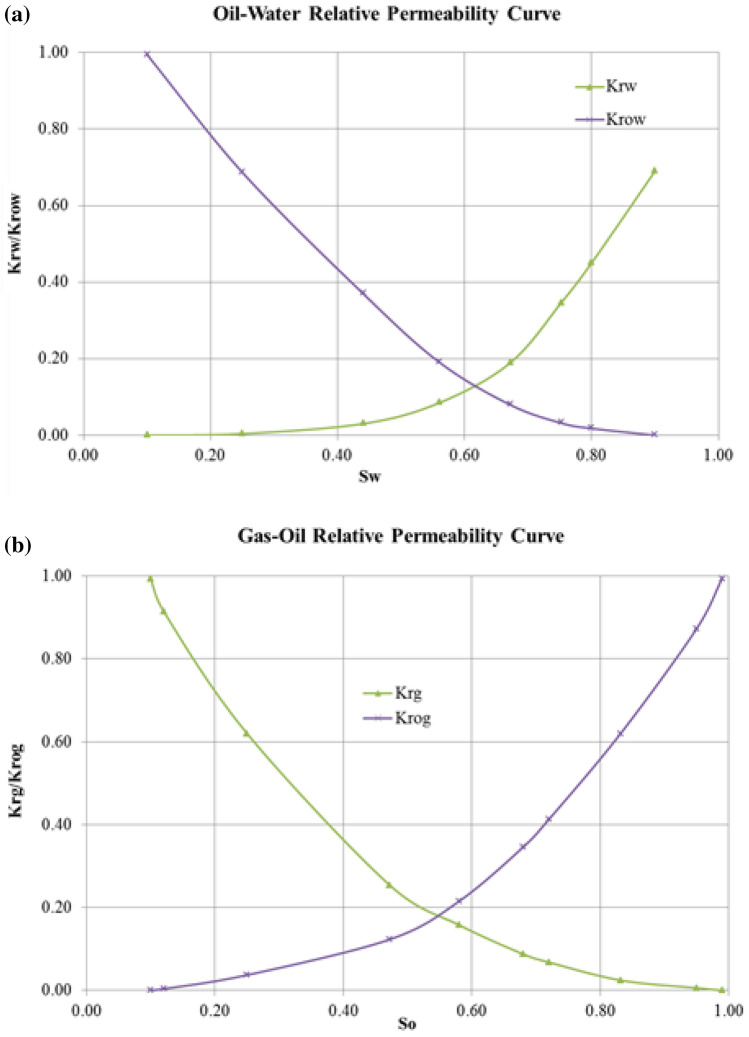
Relative permeability curves for the Canadian Athabasca bitumen for **a** oil/water, and **b** gas/oil

The porosity, absolute permeabilities, and initial fluids saturations, which are those of the typical Athabasca bitumen, are shown in Table 1. These parameters were also used in the validated models presented by Rabiu Ado (2017) and Ado (2020a). The relative permeability curves for oil/water and gas/oil are shown in Fig. 2.

**Table 1:** Porosity, absolute permeabilities, and initial fluid saturations in the reservoir

Reservoir property	Value
Porosity	0.34
Absolute vertical permeability (mD)	3450
Absolute horizontal permeability (mD)	11,500
Oil saturation, *S*_o_	0.85
Water saturation, *S*_w_	0.15
Gas saturation, *S*_g_	0.00

### Pressure, density, and temperature (PρT) properties of the bitumen

Since heavy oils and bitumen are a mixture of so many compounds, it is impractical to represent all the compounds individually. As a result, pseudo-components are used which are a cut of the bitumen, for example, over a specified boiling temperature range. In this work, and also in accordance with the validated model (Ado 2020a) that is being used in this work, two oil pseudo-components are used to represent the whole bitumen. These are called HEAV oil and LITE oil. Table 2 which is extracted from Ado (2020a) shows the critical parameters, density, molecular weight, composition, acentric factor, and normal boiling points of the pseudo-components.

**Table 2:** PVT data as reported in Ado (2020a)

Components	Split (mol%)	RMM (g/mol)	*P*_c_ (kPa)	*T*_c_ (°C)	*ρ* (kg/m^3^)	*ω*	*T*_B_ (°C)
LITE oil	36.47	170.00	2305.95	425.16	903.80	0.48	246.60
HEAV oil	63.53	878.00	1031.29	780.00	1012.07	1.45	711.00

The temperature-and-pressure-dependent *K* values needed to represent and account for vapour–liquid equilibrium and the viscosity of the individual oil pseudo-component can be found in Ado (2020a) as they are the same. Thus, it is not worth reproducing them here so as to conserve space.

### Kinetics scheme and it is parameters

In all the three models, improved modified kinetics scheme which is very well validated against experimental results and referred to as “model G” in Ado (2020a) is used together with it is Arrhenius parameters. This kinetics scheme is also fully detailed in Ado (2020c) where it is described as “split conversion kinetics scheme (SCKS)” due to the fact that the concentrations of the thermal cracking reactions’ products are heavily dependent on their selected stoichiometric coefficients. This SCKS is highly indeterminable because there are infinite number of possible combinations to obtained atom- and mass-wised balanced chemical reaction. For example, the SCKS is expressed as HEAV Oil → (*x*)LITE Oil + (*y*)Coke such that *x* and *y* as the stoichiometric coefficients can have any numbers. This, therefore, ultimately increases the number of unknowns in the reactive transport equations. As a result, and for the sake of clarity and knowledge advancement and sharing, SCKS-type schemes cannot be systematically up-scaled to study field scale scenarios and give meaningful predictions [please see Ado ([Bibr CR2][Bibr CR3]) for full details]. However, since it is very well validated and is able to provide physically meaningful predictions at laboratory scale, it is used in this study as can be seen in Table 3.

**Table 3:** Thermal cracking and combustion reactions with their Arrhenius parameters as reported in Ado (2020a)

	Frequency factor (min^−1^)	Activation energy (kJ/mol)	Heat of reaction (kJ/mol)
Thermal cracking reaction			
HEAV → 1.6 LITE + 46.6 Coke	1.50 × 10^9^	99.00	0.00
Combustion reactions			
HEAV + 80 O_2_ → 26.7 H_2_O + 68.7 CO_1.94_	1.81 × 10^11^ kPa^−1^	138.00	5.91 × 10^2^
LITE + 19 O_2_ → 14.5 H_2_O + 11.8 CO_1.94_	1.81 × 10^12^ kPa^−1^	138.00	4.91 × 10^2^
CH + 1.22 O_2_ → 0.5 H_2_O + CO_1.94_	8.60 × 10^7^ kPa^−1^	123.00	3.65 × 10^2^

Note that the other components, in addition to the two oil pseudo-components, are shown in the balanced chemical reactions in Table 3. O_2_ = oxygen gas in air; H_2_O is water vapour in the combustion gases; CH = Coke which is the carbonaceous immobile fraction responsible for operation in HTO mode; CO_1.94_ is the combination of carbon oxides (i.e. CO_2_ and CO) so that lower number of components are used to lessen the number of equations that have to be solved.

### Initial and boundary conditions

Initially inside the HP well, oil saturation is assigned a value of 0 in each model. This is justified because at the native reservoir condition, the bitumen is immobile and none of it has entered into the HP well prior to heat addition. The initial reservoir temperature and pressure are respectively 27 °C and 290 kPa which are in accordance with the experimental setting against which the model is validated (Xia and Greaves 2002). All over the combustion cell, in each model, no flow boundary condition is assigned with exception of flows through injector(s) and producer. The HP well in each model was assigned two boundary conditions, namely maximum liquid production rate of 25 cm^3^ min^−1^ and minimum bottom hole pressure of 170 kPa. The simulator uses either of the two depending on which one is violated. For the HI or 2VI well, a boundary condition of 8000 Scm^3^ min^−1^ of air injection rate in the first 190 min is assigned. Thereafter, the air injection rate was increased to 10,667 Scm^3^ min^−1^. In the case of 2VI wells, the air injection rate via each VI well is half of the total whilst in the case of the HI well, the total air injection rates were sent via the vertical arm of the well so that the air exits through the horizontal arm which has 17 perforations. In terms of pre-ignition heating, steam or electrical heater was used with appropriate boundary condition identified hereon. For models A and C, steam containing the equivalent amount of heating rate in model B (i.e. 2115 J min^−1^ per gridblock) was injected at a rate of 21.17 cm^3^ min^−1^ cold water equivalent (CWE) for a period of 28 min in each of the two models. Then, in each model, electrical heater/ignitor was used for 2 min to further increase the temperature of the inlet zone of the injectors and subsequently to initiate the combustion. With regard to heat loss, heat loss parameters are specified for the overburden and underburden. However, in all the horizontal–vertical sides (i.e. sideways areas), no heat loss is assumed. Since no matter crosses the overburden and underburden, the heat loss via them will be due to conduction only.

### Optimum grid block sizes selection

For the sake of completeness, sensitivity of the simulation results against grid block sizes is investigated thoroughly as exemplified by study in Rabiu Ado (2017). In total, 38,000 grid blocks (i.e. for reservoir and discretised wellbore combined) are found to be sufficient in terms of capturing the full physics of the combustion front and taking the optimum computational time to do so. The total computational time is 2 h, 54 min when dynamic gridding was not used. However, it is found that all the key performance parameters for studying the THAI process are insensitive to the use of dynamic gridding. Therefore, it is worth pointing out here that the dynamic grid block refinement and de-refinement functionality in the CMG STARS (i.e. DYNAGRID) was used so that savings were made in terms of avoiding unnecessary calculations that would lead to taking more computational time. As a result, the computational time for each of the three models is 1 h, 58 min.

## Results and discussions

In order to fully use the key economic and safety indicators to decisively conclude the best or optimum well configurations together with best or optimum pre-ignition heating method, the following parameters as predicted by the models, which are derived from the validated model, are presented, analysed, discussed and compared:

(i) Oil production rate,
(ii) Cumulative oil produced and thus recovered,
(iii) Peak temperature, and
(iv) Produced oxygen concentration

No two- or three-dimensional profiles are considered here because they have already been analysed and discussed in multiple previous studies.

### Oil production rate

To reiterate, model A consists of two vertical injection wells with a horizontal producer well (2VIHP) configured in a staggered line drive (SLD) pattern. In it, steam is the method of PIHC. For models B & C, the horizontal injector well with a horizontal producer well (HIHP) are configured in an SLD arrangement. Electric heating is used for the PIHC in model B whilst steam is used in model C. Given the afore-explained, models A and C are compared first since they both use steam for pre-ignition heating and that their only differences in terms of setting are the wells geometry and configuration. Onwards, models B & C are compared as they both have the same wells geometry and arrangements but differ in the method of pre-ignition heating.

#### Pre-ignition heating period

**Fig. 3:**
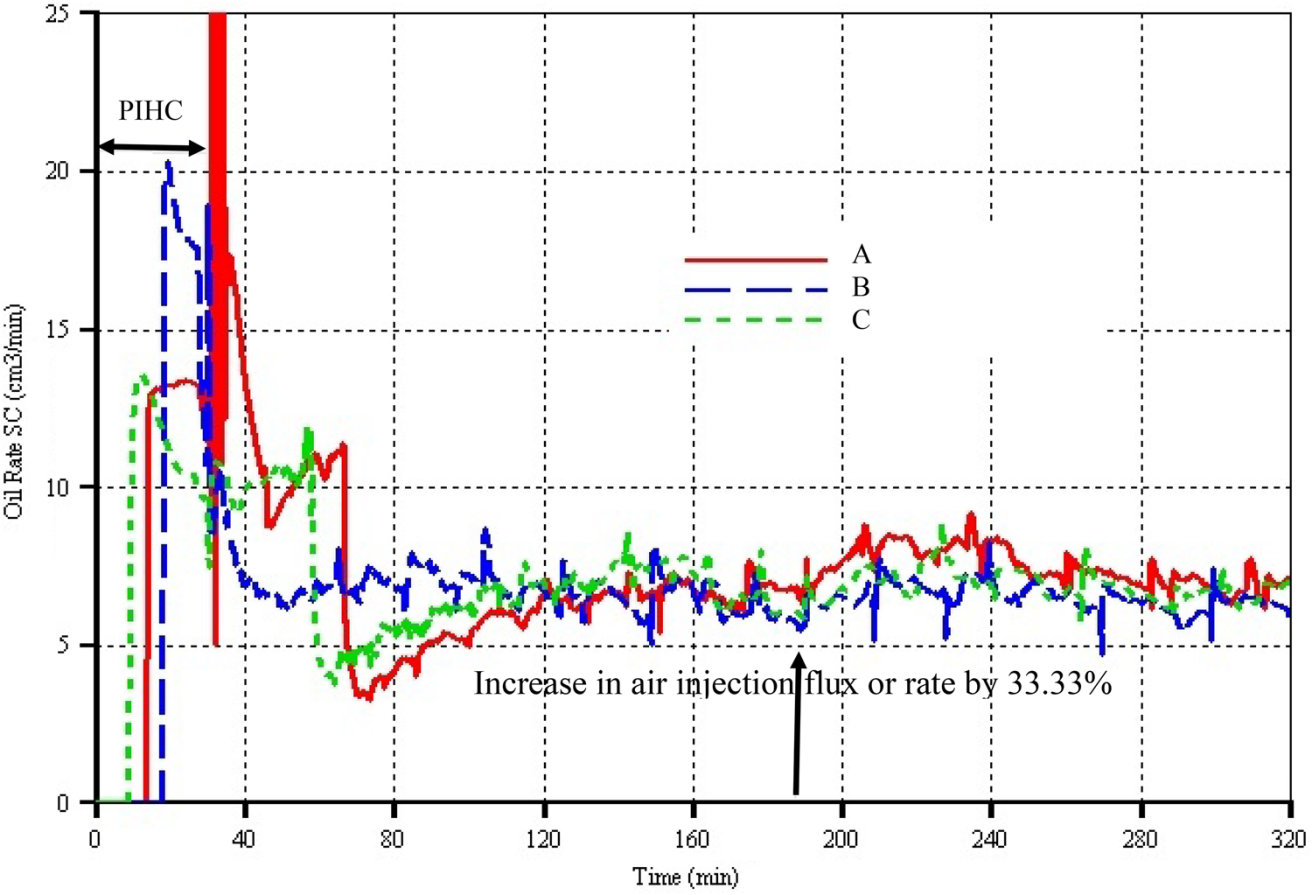
Oil production rates versus time for the three models. The arrow pointing vertically upward indicates the time when the air injection rate was increased by 33.33%

**Fig. 4:**
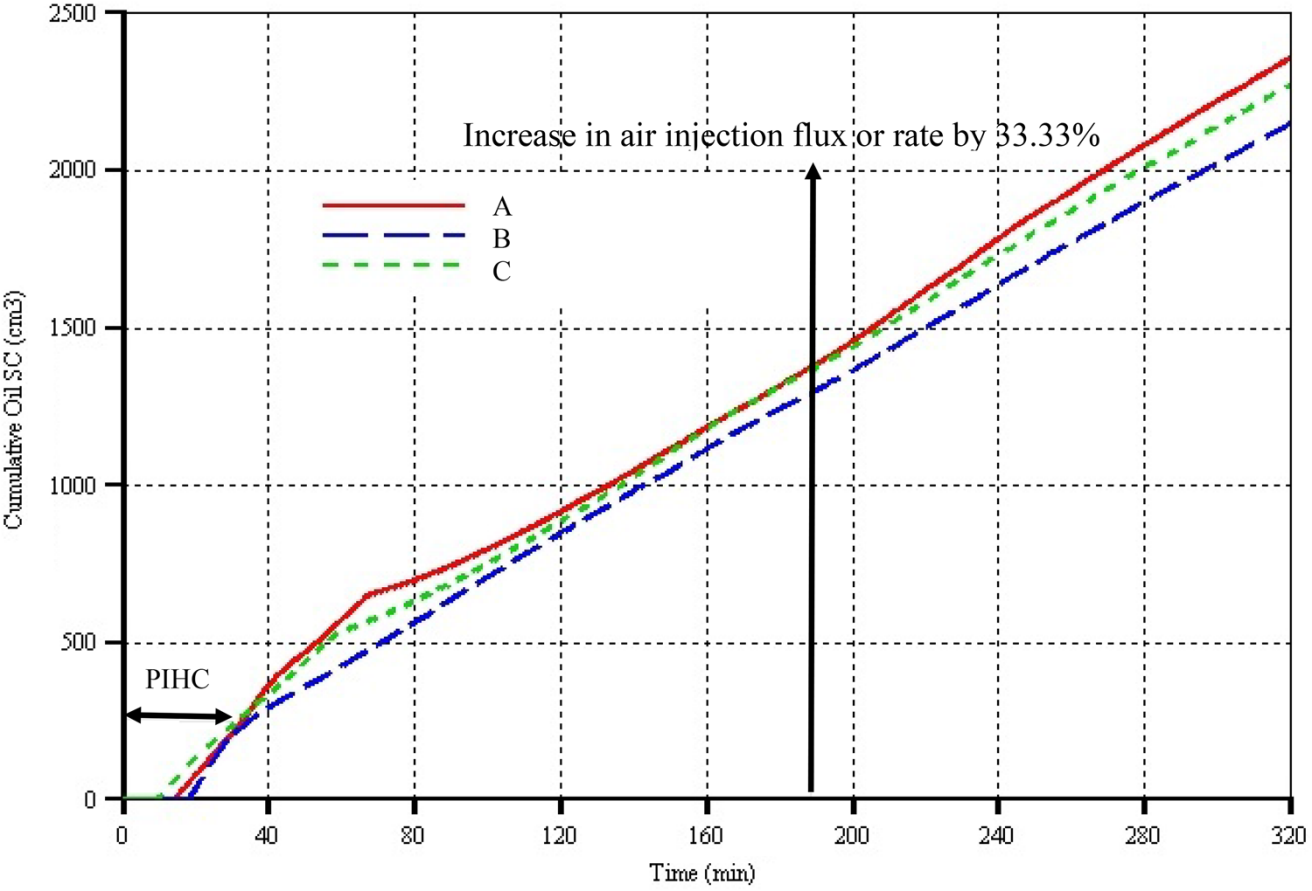
Cumulative oil production curves versus time for the three models. The arrow pointing vertically upward indicates the time when the air injection rate was increased by 33.33%

Due to the spread of HI well in model C and thus having one of the perforations directly in the same plane as the HP well, oil production began just 10 min after the start of the PIHC. In the case of model A however, there is a delay by 5 min (i.e. oil production began in model A 15 min after the initiation of PIHC) which is due to the fact that the mobilised oil from around the shoe of each VI well has to travel both vertically downward and laterally towards the mid-plane where the HP well is located. This implies that model A should take a little longer before oil production began during the start-up. Both models A & C have their initial oil production rates instantaneously peaking to around 16 cm^3^ min^−1^ (Fig. 3). In model A, the rate remains roughly at that value until steam injection was stopped at 28 min and electrical heater/ignitor was used to rise the temperature to higher value just prior to air injection. Thereafter, due to absence of large momentum transfer to the already mobilised oil, the oil production rate dropped to a lowest value of 5 cm^3^ min^−1^ during the last 2 min of electrical heating just before ignition. However, in the case of model C, the oil production rate dropped steadily from 16 cm^3^ min^−1^ at 10 min to 9 cm^3^ min^−1^ by 28 min (Fig. 3). As the steam pre-ignition was stopped and electrical heater/ignitor put on for the remaining 2 min of the PIHC, the oil rate in model C declined further to around 7.5 cm^3^ min^−1^. Therefore, in both models A & C, fluid communication was successfully established during the PIHC. To check whether some steam is lost via channelling into the HP well especially in model C, the cumulative oil production curves were compared at 30 min. It is found that both models A & C curves overlap each other around that region (see Fig. 4) thereby implying that the same cumulative quantity of oil is produced even though that the quantity mobilised in model A is evidently greater than that in model C as we shall see in the next section.

Now comparing models B & C, it can be seen from Fig. 3 that oil production in model B began 20 min after the commencement of electrical heating, implying that it lags model C by 10 min. This large lag can be attributed to two things: (i) heat transfer from the electrical heating is dominantly by conduction especially at early stages before hot mobilised oil mixes with the cold oil located below the electrically heated zone and near the toe of the HP well, and (ii) absence of pressure force or momentum transfer from other fluids like steam causes the mobilised hot oil to reach the HP well due to mainly gravity drainage. The oil production rate in model B began by shooting to 20.5 cm^3^ min^−1^ before descending slightly slowly, reaching 17.5 cm^3^ min^−1^ at 28 min (Fig. 3). Thereon, it further declined but this time sharply to around 11 cm^3^ min^−1^ at 30 min (i.e. the end of the PIHC period). It is believed that the decline is caused by the fact that all the mobilisable oil in direct contact with the electrical heater/ignitor has already been displaced and drained and that the to-be-mobilised oil in the cold oil zone is thus not in direct contact with the electrical heater/ignitor. Over the 10 min of oil production in the PIHC phase in model B, its oil rates are higher, sometimes even twice, than those in model C. That means, the higher oil rates in model B could counter and balance the oil produced in model C. This is exactly the case as reflected in the cumulative oil production curves where, by 30 min, cumulatively, the same quantity of oil is recovered in both models (see Fig. 4). Therefore, in terms of start-up, in all the three models, it can be concluded that the same quantity of oil is cumulatively recovered by the end of the PIHC (Fig. 4). However, it is clearly determined that realisation of return on investment will be delayed by days or even months in real field operations where both models A & B start-up methods and wells configurations are used. The best model in terms of early production is model C which is followed by A which in turn is followed by model B. The latter is the worst because the heat transfer is mainly by conduction, and consequently in the field, it is expected that longer than economically profitable period will be taken to establish fluid communication between the wells if model B settings are to be used. Therefore, during start-up, ultimately, steam injection instead of electrical heating should be used for the pre-ignition heating. But, this is just about the information from the start-up. The question that ought to be asked and answered is that: what about maintenance of optimal operations throughout the lifetime of the project?

#### Combustion period

**Fig. 5:**
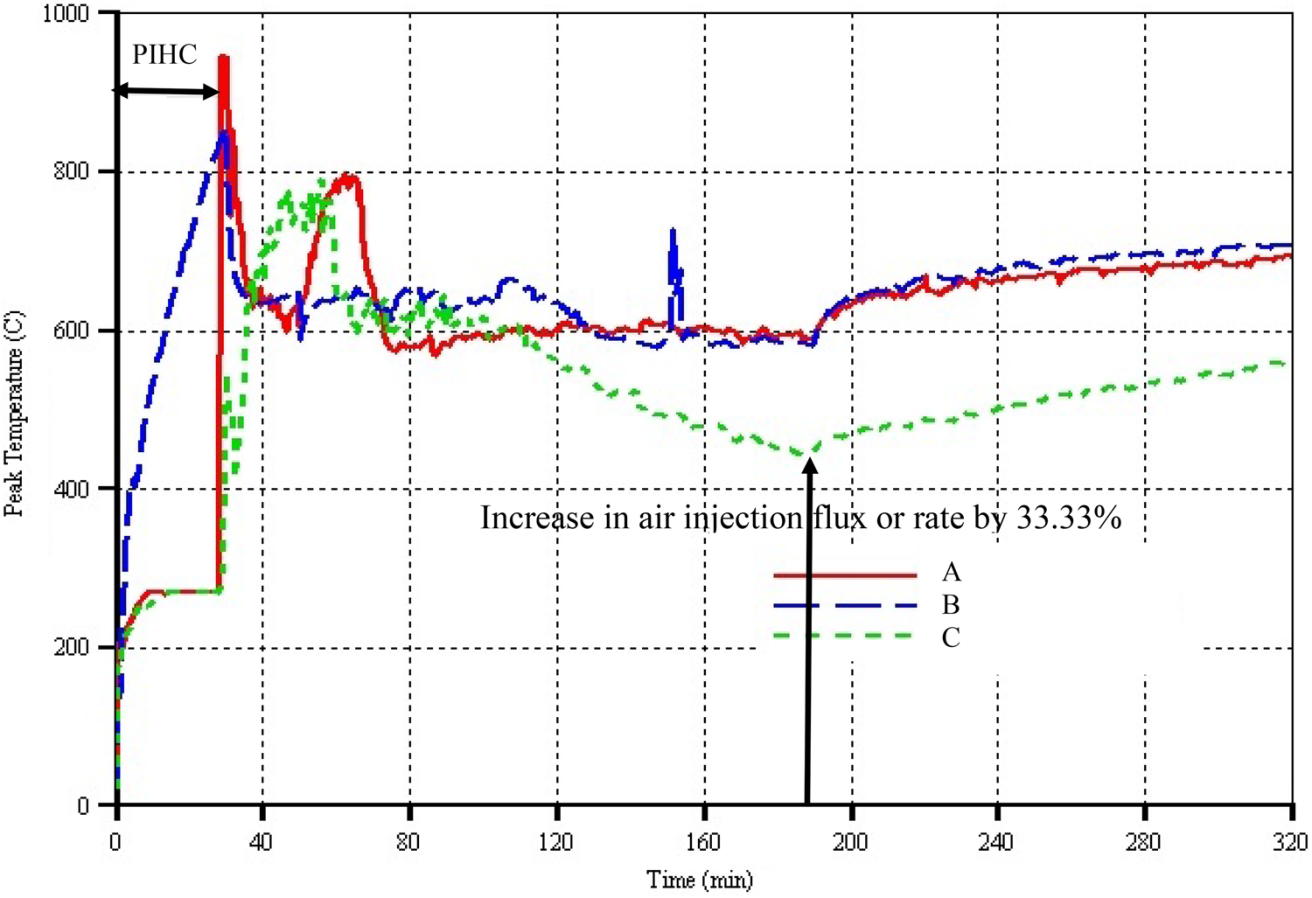
Peak temperatures versus time for the three models. The arrow pointing vertically upward indicates the time when the air injection rate was increased by 33.33%

Upon electrical heating for the 2 min (i.e. from 28 to 30 min for models A & C) and injection of air together with electrical ignition in all the models, instantaneous increase in oil production rates can be seen in Fig. 3, ranging from very steep, moderately sharp, and slightly abrupt in models A, B, and C respectively. These took place for the reason that the already mobilised partially upgraded oil that has not drained into the HP well was swept and forced to drain due to gas momentum transfer. However, the sudden rapid increase in the oil rate in model A is peculiar and merits further attention as it lasts for around 5 min (i.e. from 30 to 35 min). This is caused due to the fact that both laterally to the left of VI well 1 and to the right of VI well 2, there were already mobilised oils that the relatively very low steam injection rate (i.e. 10.585 cm^3^ min^−1^ per VI well) could not reach to sweep them. Similarly, for model B, the already mobilised oil that did not drain due to absence of push from another fluid was swept by the large volume of gas leaving the combustion zone. Furthermore, for both models A & B, there were small concentrations of the immobilised oil pseudo-component left surrounding the inlet zone of the combustion cell. Partly, also that is why the oil production rates increased correspondingly with the increase in their peak temperatures (see Fig. 5) respectively. For model C, the increase in oil production rate was comparatively smaller. This is as a result of the fact that low volume flow rate of steam enters the combustion cell via each of the 17 perforations in the horizontal arm of the HI well. Moreover, larger area of the reservoir is affected by the steam due to convection being the dominant mechanism of heat transfer thereby causing very high concentration of heavier fractions to remain around the HI well. These resulted in them consuming more energy to effect thermal cracking and at the same time for the combustion to be sustained (see Fig. 5 where the peak temperature of model C is the lowest between 30 and 35 min).

Beyond 35 min model A and C predicted decrease, then increase, and then later decrease in the oil production rates until they reach minimums of 3.5 cm^3^ min^−1^ at around 75 min and 4 cm^3^ min^−1^ at around 65 min for models A and C respectively. Model B predicted decrease in the oil production rate over 35 to 50 min period, touching a minimum value of 6.5 cm^3^ min^−1^ at the latter time (Fig. 3). This dynamic or transient behaviour is associated with the injected air cooling effect in which there is competition between using the high-temperature oxidation (HTO) heat of the combustion zone for upstream heating of the entering air or for downstream heating of the different physicochemical zones to continue to achieve thermal cracking upgrading and mobilisation of cold oil. Past the minimum points in each model, there was steady increase in the oil rates in models A & C, with that of the former below that of the latter between 70 and 120 min. For model B, past the minimum oil production rate to around 180 min, the oil rate steadied out at an average value of ≈ 7 cm^3^ min^−1^ before slightly decreasing and then increasing slowly and flattening at an average of ≈ 7 cm^3^ min^−1^ again up to the end of the dry combustion period (Fig. 3). Models A & C have similar trends as that in B with the exception of oil production rate in model A which lies above those of B and C from 190 to 320 min. Likewise, over the same time range, that of model C lies above that of model B. In general, it can be concluded that steady state is achieved from around 110 min in each model, and that although all the models dynamically responded to the increase in air injection rate, no profitable or appreciable increase in oil rates is achieved due to the increase in the air injection flux. This finding has been observed from Kerrobert field data in which Wei et al. (2020b) found nonlinear relationship and in some cases no relationship between air injection rate and oil production rate. Therefore, optimum air injection rate must be determined so that unnecessary spending of operating cost is avoided. Also, it is worth pointing that any required increase in air injection rate must only be performed after the combustion front is fully established and steady fluids productions are realised. Clearly, and as reflected in the cumulative oil production curves of Fig. 4, model A has higher oil production rates after the increase in air flux, and thus, it can be concluded that for long-term projects, model A settings and wells configuration should be used. However, this is not decisively conclusive because there are other critical parameters that must be analysed.

### Cumulative oil production

In each model, roughly 250 cm^3^ of oil is recovered by the end of the PIHC. This implies that the same quantity of energy was added and the same reservoir volume was swept by the pre-ignition heat. However, as mentioned earlier, the oil mobilisation inside the combustion cell is model dependent and at the start of ignition, the remaining unproduced mobilised oil was forced out in each model. In fact, the large quantity of it can be seen to have caused the model A curve (Fig. 4) to have bigger slope between 40 and 80 min. Thereafter to around 145, curve A lies above those of models B & C. Between 145 and 200 min, curves A and C overlapped before then diverging, with curve A lying above C for the rest of the dry combustion period of 320 min. That convergence can be ascribed to the fact that from 80 to 160 min, oil production rates in C are greater than in A which lead to curve C progressively moving towards A until the reverse has happened. However, this behaviour of model C is counter intuitive especially when the fact that from 110 min onwards, the peak temperature of model C lies below that of model A by up to 150 °C (Fig. 5). This finding has placed question mark over the use of peak temperature to adjudge the overall quality of burning in the combustion front. Therefore, one of the major findings of this work is the fact that peak temperature does not, in certain situations, provide the measure of the efficient propagation of combustion front. This is because the simulator or even thermocouple in practical settings gives the highest value of the temperature at one single point relative to all remaining other points. As an example, consider the combustion front to have fingers such that at the leading edge of one finger, it encounters very large concentration of coke that presence of sufficient concentration of oxygen leads to large quantity of heat generation which will of course be reflected by the change in the temperature at that location. Therefore, the use of peak temperature will not tell us anything about other locations where the burning is in reality barely detectable. To summarise, in certain situations, the oil production rates, and thus the cumulative oil production, are not proportional to the peak temperature. From 30 to 320 min, curve B lies below those of models A & C thereby implying that oil production rates in the former are generally lower than in the latter two models. This is despite the fact that model B’s peak temperature is mostly higher than that of model C by up to 150 °C. Overall, a higher cumulative oil recovery of 2350 cm^3^ is obtained in model A which is greater than those of B and C by 9.6% and 4.3%, respectively. Therefore, based on this, it can be concluded that model A is more efficient and thus profitable.

### Peak temperature

From the peak temperature curves (Fig. 5), it can clearly be seen that at 28 to 30 min, both models A and C have very large increase in peak temperature, with that of the former being substantially higher, reaching up to 950 °C compared to the 530 °C in the latter. For model B, the peak temperature increased comparatively gradually, reaching 850 °C by the end of 30 min of PIHC. This is because conduction was the dominant mechanism of heat transfer during the PIHC of model B. On gas injection and just before combustion is started, an injected air cooling effect can be observed in all models. This finding has been observed in the field where heat consumption could not momentarily be balanced by the heat being generated (Wei et al. 2020b). Once combustion is started, the peak temperature of model A continues to decrease until around 50 min when it raised sharply reaching a new high value of 800 °C at 65 min before declining again and steadying out at an average of 600 °C until just before increase in air injection flux.

**Fig. 6:**
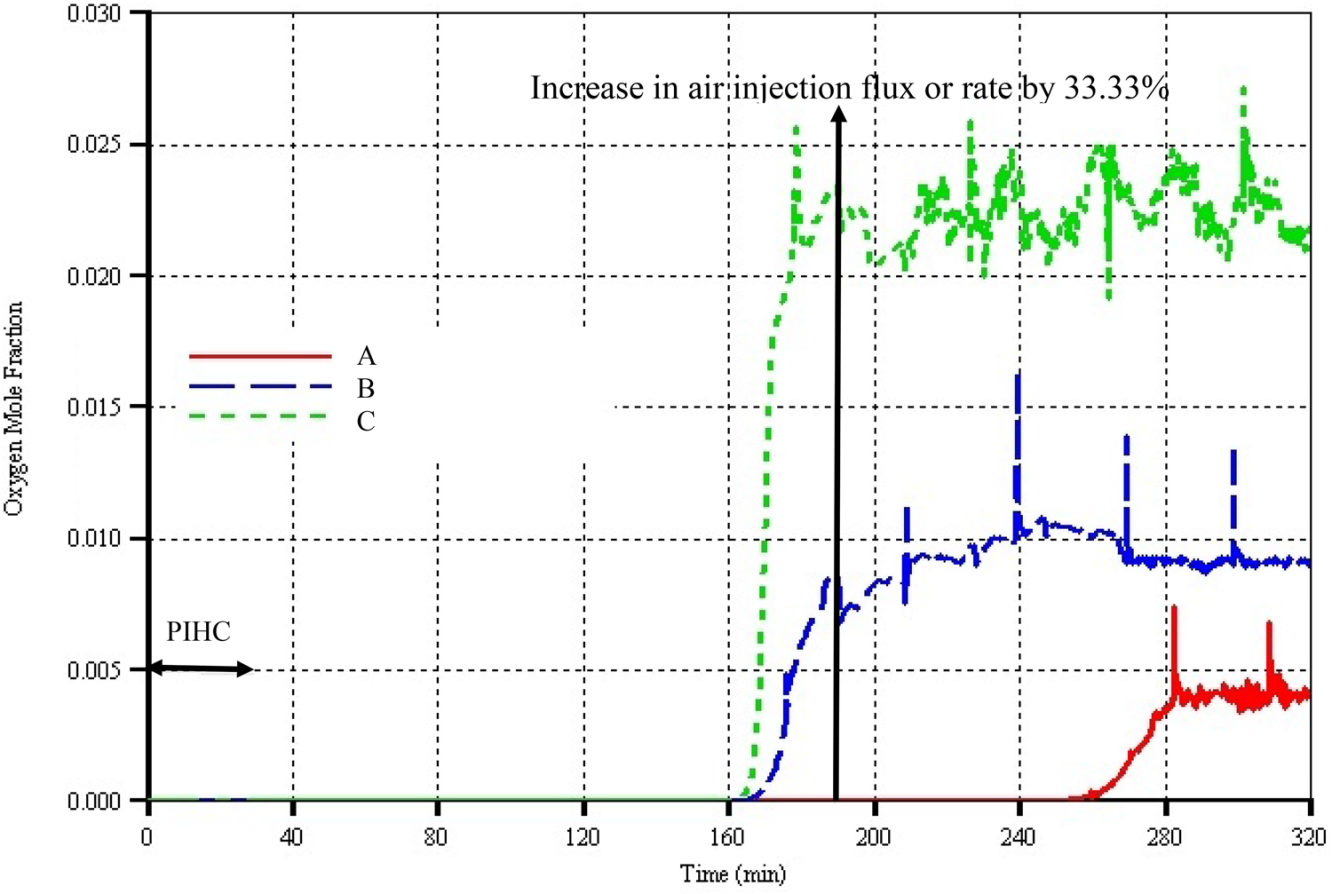
Produced oxygen mole fraction curves versus time for the three models. The arrow pointing vertically upward indicates the time when the air injection rate was increased by 33.33%

In model C, just immediately combustion was started, there is sharp increase in peak temperature, climbing to 800 °C at 58 min before falling rapidly and steadying out at an average of 650 °C until 110 min. Beyond that, the peak temperature declines steadily, hitting 440 °C at 190 min before picking-up again after increase in air injection flux. In model B, the peak temperature more or less steadied out at an average value of 640 °C until 120 min. Thereafter, it declined slightly before settling at around 600 °C up to 190 min. Just before 160 min, a spike in the peak temperature of model B, which does not appear in models A and C, indicates the time when the combustion front has reached the toe of the horizontal producer (HP) well where there was a very high concentration of coke that lead to higher rate of heat generation and hence higher peak temperature. Once the coke at the toe was fully consumed, which appeared to have taken 15 min (Figs. 5, 6), oxygen production began. This is another major finding in this study which is similar to experimental observation (Xia and Greaves 2002). In model A, it simply means that the combustion did not reach the toe of the HP well from either of the two vertical injectors due to the lateral distance separating them from the toe of the HP well. Similarly, in C, it appears that although the combustion front has reached the toe of the HP well, the steaming during the PIHC has washed out most of the oil there to the extent that the concentration of the coke is generally lower and thus did not result in spike formation on the peak temperature curve. Like in model B, oxygen production in model C began at around 163 min (Fig. 6), indicating that indeed, the combustion front has reached the toe and has started to propagate along the HP well.

Immediately when air injection flux is increased by a factor of $\frac{4}{3}$, the peak temperatures in models A and B increased gradually whilst overlapping each other before slightly diverging with model B laying above A until the end of the dry combustion time (i.e. 320 min). The peak temperature in model C also trends similarly whilst laying below those in A and B by up to 150 °C until the end of the dry combustion time. In general, what makes the peak temperature in model C for most of the combustion time to lie below those of A and B is additionally the fact that coke concentrations ahead of the combustion front are generally lower due to significant displacement of oil especially between 75 and 160 min (Fig. 3). Therefore, that makes the rate of the HTO reaction and thus rate of heat generation lower, leading to lower peak temperatures. Another reason could be due to small amount of oxygen channelling from the toe and heel of the horizontal injector (HI) well respectively. However, the question that might be asked is about the delay in the start of oxygen production if it indeed was channelling from around 110 min. And the answer is the fact that it had to displace water in the cold oil zone before it can reach the heel of the HP well and get produced from there.

In conclusion, although it is now discovered that the peak temperature cannot in all settings tell how healthy a combustion front is, it has revealed that model A does indeed have far more stable, safer, and efficient combustion front propagation due to the maintenance of very high peak temperatures throughout.

### Produced oxygen concentration

To reiterate, in models B and C, oxygen production began at 163 and 165 min respectively (Fig. 6). However, in the latter, it initially jumped to 2.5 mol% before it oscillates between 1.9 and 2.75 mol% for the rest of the combustion period. The oscillatory nature of the produced oxygen could be attributed to variabilities of coke concentrations along and around the HP well even though at least three reasons for the course of oxygen production in model C have been identified and explained in the preceding section. These concentrations are high enough to cause corrosion in the long term or even to burn inside the HP well which could lead to explosion. These findings are in accordance with conventional ISC field data (Sharma et al. 2021).

In the case of model B, oxygen production is due to propagation of combustion front along the HP well only and since it has higher coke concentrations and peak temperature, the maximum concentration of the produced oxygen is just a little over 1 mol% with the exception of five sharp spikes that might have been caused to appear due to the very small time step sizes during the numerical simulations. This is unlike in physical experiments where sampling rate is lower. It is worth noting that at around 270 min, the concentration of the produced oxygen in model B has dropped to 0.9 mol% and remained steady thereafter (Fig. 6). This could be an indicator that in the long run, breakthrough can only take place when oil production rates become uneconomical due to the combustion front nearing the heel of the HP well. In model A, oxygen production began at around 260 min with the maximum concentration of just around 0.4 mol% (Fig. 6), and it appears to have reached steady state value by remaining constant till the end of the combustion time. This concentration is very low and is highly unlikely that it will pose safety or operational problems. It might be caused by creation of very narrow channel due to presence of very low or even zero concentration of coke in that zone.

To conclude, model A is by far more efficient in terms of burning quality of combustion, more stable, and safer in terms of concentration of oxygen in the produced gas stream. This is then followed by model B. Model C is unstable, unsafe, and inefficient in both the short and long terms.

## Conclusion

The THAI process is yet to be technically and economically well proven despite pilot and semi-commercial operations. Some studies concluded using field data that THAI is a low-oil-production-rate process. However, no study has thoroughly investigated the simultaneous effects of start-up methods and wells configuration on both the short and long terms stability, sustainability, and profitability of the process. Using THAI validated model, three models having a horizontal producer well arranged in a staggered line drive configuration with the all injector wells are simulated using CMG STARS. Model A has two vertical injectors via which steam was used for pre-ignition heating, and models B and C each has a horizontal injector via which electrical heater and steam were, respectively, used for pre-ignition heating. It is found that during start-up, ultimately, steam injection instead of electrical heating should be used for the pre-ignition heating especially with the two vertical injectors. Clearly, it is shown that model A has higher oil production rates after the increase in air flux and also has a higher cumulative oil recovery of 2350 cm^3^ which is greater than those of models B and C by 9.6% and 4.3% respectively. Thus, it can be concluded that for long-term projects, model A settings and wells configuration should be used. Although it is now discovered that the peak temperature cannot in all settings tell how healthy a combustion front is, it has revealed that model A does indeed have far more stable, safer, and efficient combustion front burning quality and propagation due to the maintenance of very high peak temperatures of mostly greater than 600 °C and very low concentrations of produced oxygen of lower than 0.4 mol% compared to up to 2.75 mol% in model C and 1 mol% in model B. Conclusively, since drilling of, and achieving uniform air distribution in horizontal injector (HI) well in actual field reservoir are costly and impracticable at the moment, and that electrical heating will require unphysically long time before mobilised fluids reach the HP well as heat transfer is mainly by conduction, these findings have shown decisively that the easy-and-cheaper-to-drill two vertical injector wells configured in a staggered line drive pattern with the horizontal producer well should be used, and steam is thus to be used for pre-ignition heating.
